# Peace, Liberty, Mycobacteria, and Tuberculosis Mortality

**DOI:** 10.3201/eid2403.AC2403

**Published:** 2018-03

**Authors:** Terence Chorba

**Affiliations:** Centers for Disease Control and Prevention, Atlanta, Georgia, USA

**Keywords:** art science connection, emerging infectious diseases, art and medicine, about the cover, Antonio de Francisci, US Silver Peace Dollar, Peace, Liberty, Mycobacteria, and Tuberculosis Mortality, tuberculosis, tuberculosis and other mycobacteria, Mycobacterium tuberculosis, bacteria, sulfamidochrysoidine, respiratory infections

**Figure:**
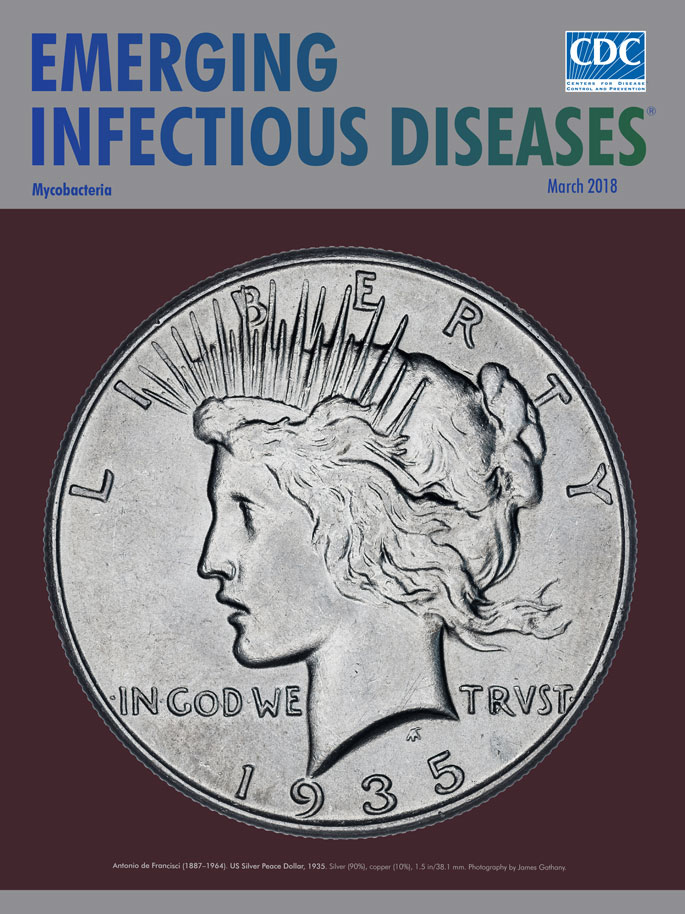
Antonio de Francisci (1887−1964). US Silver Peace Dollar, 1935. Silver (90%), copper (10%), 1.5 in/38.1 mm.

In 1935, Gerhard Domagk and Josef Klarer, working with dyes at the Bayer Institute of Pathology and Bacteriology, published the results of several clinical investigations of sulfamidochrysoidine. This antibacterial drug was the first of the sulfonamide-containing or related products that transformed approaches to treatment of infection and heralded the antibiotic era. Before that, the only antimicrobials available were the arsenicals (arsphenamine and neosalvarsan), which were used to treat syphilis. Sulfamidochrysoidine, produced under the trademark name of Prontosil, was demonstrated to be effective for treating streptococcal and staphylococcal infections. In 1939, Domagk was recognized with the Nobel Prize in Physiology or Medicine; although the Third Reich forced him to decline the award, he subsequently received it in 1947.

The year 1935 also was the final one in which a US dollar made predominantly of silver (silver dollar) was minted for general circulation. The coin was first issued in 1921. Its design earned it the name “Peace Dollar”; its reverse side portrays an American bald eagle with an olive branch in its talons, intended to celebrate the long-lasting peace that was to have followed the Great War, World War I. The bird is at rest, its wings folded, perched on a craggy rock above the word “Peace,” facing the dawn’s rays rising beyond distant hills.

The front side of the coin depicts the profile of the head and neck of the goddess Liberty, a personification who had been represented on coins in ancient Greece as Eleutheria and in ancient Rome as Libertas. A departure from previous and more staid US representations of Liberty, the Peace Dollar goddess has hair pulled into a bun with stray locks loose and flowing, and wears a radiant tiara, likened to the sun god, Helios, as seen in depictions of rulers on coinage back to the third century bce.

From the coin’s outset, the identity of the Liberty model was disputed. Some experts contended that the model was Maria Teresa Carafelli, wife of the coin’s designer, Antonio de Francisci, who had immigrated to America in 1905 and studied under several sculptors known for their designs of US coins of other denominations: James Fraser (the Buffalo Nickel, 1913–1935), Hermon MacNeil (the Standing Liberty Quarter, 1916–1930), Adolph Weinman (the Mercury Dime, 1916–1945), and Augustus Saint-Gaudens (the $20 Double Eagle, 1907–1933). However, de Francisci maintained that the visage was a “composite” face that “typified something of America.” In her later years, Carafelli held that she was not the model for Liberty but “just an accessory.”

The other rumored model for Liberty was a British-born actress, Maryland Morne, who was reputed to have won a contest for the job. Coincidentally, Morne died of tuberculosis in the same year that the minting of the Peace Dollar ceased; in 1935, newspaper notices of her death and funeral unequivocally declared her the “Peace Dollar Girl.” Unfortunately, the newly discovered antimicrobial activity of sulfamidochrysoidine included little if any activity against *Mycobacterium tuberculosis.* It was not until 1944 that Albert Schatz, Elizabeth Bugie, and Selman Waksman identified streptomycin produced by *Streptomyces griseus* as the first antimicrobial with bactericidal activity against mycobacteria.

The understanding that neither poverty nor genetics was the primary cause of tuberculosis came after the discovery of the transmissible organism, *M. tuberculosis,* announced by Robert Koch, on March 24, 1882; for this discovery, he was duly recognized with the Noble Prize for Physiology or Medicine in 1905. In 1900, the reported US mortality rate from tuberculosis was in excess of 200 deaths per 100,000 population. Through the years that the Peace Dollar was minted, recognition of an infectious etiology contributed greatly to decreases in tuberculosis morbidity and mortality in the United States, even in the absence of antimicrobial drugs, as attention focused on containment and exclusion of persons symptomatic with disease from close contact with others. From the beginning of Peace Dollar coinage in 1921 to its end in 1935, the US mortality rates from tuberculosis decreased from 99.4 deaths per 100,000 population to 55.0 deaths per 100,000 population. Today, the US mortality rate for persons for whom tuberculosis was indicated as a cause of death is <0.01 deaths per 100,000 population, owing largely to understanding the need for respiratory precautions to disrupt transmission but owing also to appropriate use of antimicrobial drugs to treat active disease and latent infection, and to contact investigation and screening of persons in high-risk populations.
